# DC-Derived Exosomes for Cancer Immunotherapy

**DOI:** 10.3390/cancers13153667

**Published:** 2021-07-21

**Authors:** Yi Yao, Chunmei Fu, Li Zhou, Qing-Sheng Mi, Aimin Jiang

**Affiliations:** 1 Center for Cutaneous Biology and Immunology, Department of Dermatology, Henry Ford Health System, Detroit, MI 48202, USA; yyao1@hfhs.org (Y.Y.); cfu1@hfhs.org (C.F.); lzhou1@hfhs.org (L.Z.); 2 Immunology Program, Henry Ford Cancer Institute, Henry Ford Health System, Detroit, MI 48202, USA

**Keywords:** dendritic cells, exosomes, vaccines, plasmacytoid DCs, cancer immunotherapy

## Abstract

**Simple Summary:**

Dendritic cells (DCs)-based cancer vaccines have not succeeded in generating significant clinical responses despite their capacity to induce host anti-tumor CD8 T cell immunity, and one major hurdle is tumor-mediated immunosuppression. Exosomes are nano-sized inert membrane vesicles derived from the endocytic pathway that play a critical role in intercellular communication. DC-derived exosomes (DCexos) additionally carried MHC class I/II (MHCI/II) often complexed with antigens and co-stimulatory molecules, thus capable of priming antigen-specific CD4 and CD8 T cells. Indeed, vaccines with DCexos have been shown to exhibit better anti-tumor efficacy in eradicating tumors compared to DC vaccines in pre-clinical models. Coupled with their resistance to tumor immunosuppression, DCexo-based cancer vaccines have been heralded as the superior alternative cell-free therapeutic vaccines over DC vaccines, and have now been tested in multiple clinical trials. In this review, current studies of DCexo cancer vaccines as well as potential future directions will be discussed.

**Abstract:**

As the initiators of adaptive immune responses, DCs play a central role in regulating the balance between CD8 T cell immunity versus tolerance to tumor antigens. Exploiting their function to potentiate host anti-tumor immunity, DC-based vaccines have been one of most promising and widely used cancer immunotherapies. However, DC-based cancer vaccines have not achieved the promised success in clinical trials, with one of the major obstacles being tumor-mediated immunosuppression. A recent discovery on the critical role of type 1 conventional DCs (cDC1s) play in cross-priming tumor-specific CD8 T cells and determining the anti-tumor efficacy of cancer immunotherapies, however, has highlighted the need to further develop and refine DC-based vaccines either as monotherapies or in combination with other therapies. DC-derived exosomes (DCexos) have been heralded as a promising alternative to DC-based vaccines, as DCexos are more resistance to tumor-mediated suppression and DCexo vaccines have exhibited better anti-tumor efficacy in pre-clinical animal models. However, DCexo vaccines have only achieved limited clinical efficacy and failed to induce tumor-specific T cell responses in clinical trials. The lack of clinical efficacy might be partly due to the fact that all current clinical trials used peptide-loaded DCexos from monocyte-derived DCs. In this review, we will focus on the perspective of expanding current DCexo research to move DCexo cancer vaccines forward clinically to realize their potential in cancer immunotherapy.

## 1. Dendritic Cells, Anti-Tumor Immunity and Dendritic Cell-Based Cancer Vaccines

As the sentinel of the immune system, DCs play a central role in bridging innate and adaptive immune responses [1]. Known as the most potent professional antigen presenting cells (APCs), DCs initiate all adaptive immune responses by uptaking, processing, and presenting antigens including tumor antigens to activate naive antigen-specific CD4 and CD8 T cells [2,3]. Since their identification in 1973 [4], intensive studies have shown that DCs are heterogeneous populations comprising several subsets distinguished by their development, phenotype, localization, and functional specialization [5,6,7,8,9]. DCs originate in bone marrow from progenitors called common myeloid progenitors (CMPs). In the presence of transcription factor Nur77, CMPs differentiate into monocytes which can further differentiate into monocyte DCs (MoDCs) under inflammatory conditions [8]. Alternatively, CMPs differentiate into macrophage/DC progenitors (MDPs) that give rise to common DC progenitors (CDPs), which then differentiate into two major DC subsets: classical/conventional DCs (cDCs) and plasmacytoid DCs (pDCs) [5,7,8,9,10,11]. Murine cDCs consist of two subtypes currently described as cDC1s (CD11b^-^, type 1 cDCs) and cDC2s (CD11b^+^, type 2 cDCs), and their human counterparts are CD141^+^ DCs (also known as BDCA3^+^) and CD1c^+^ DCs (also known as BDCA1^+^), respectively [12]. These two subtypes of cDCs differ in their transcriptional factor dependency, function, and phenotypes. cDC1 cells include lymphoid tissue CD8α^+^ cDC1s and non-lymphoid tissue CD103^+^ cDC1s [13]. cDC1 cells depend on interferon regulatory factor 8 (IRF8) and basic leucine zipper transcriptional factor ATF-like 3 (Batf3) for their development, and are specialized in presenting internalized antigens bound to MHCI to CD8 T cells in a process termed cross-presentation [13,14]. cDC2s depend on interferon regulatory factor 4 (IRF4) and represent a heterogeneous population with enhanced MHCII antigen presentation that preferentially activate CD4 T cells [15,16,17,18]. On the other hand, pDCs are a multifunctional population best known for their specialized ability in producing and secreting large amount of type I interferons (IFNIs) [19,20,21]. pDCs also express high level of IRF8 similar to cDC1s, but depend on the E2-2 transcription factor for their development from CDPs in both mice and humans [22]. Besides MDPs, recent studies have shown that pDCs also develop from lymphoid progenitors with distinct function from their MDP-derived counterparts [23].

Cross-priming, a process which DCs prime CD8 T cells following cross-presentation of exogenous antigens onto MHCI [24,25], plays a critical role in inducing anti-tumor CD8 T cell immunity as well as mediating CD8 T cell tolerance (cross-tolerance) [26,27,28,29]. In fact, the ability of DCs to cross-present tumor-associated antigens onto an MHCI molecule to prime CD8 T cells is the foundation of the Cancer-Immunity cycle proposed by Chen and Mellman [30]. Exploiting DCs’ function to potentiate host anti-tumor immunity, DC-based vaccines have become one of the most widely-used cancer immunotherapies [7,8,31,32,33,34]. However, DC-based vaccines, the vast majority of which make use of monocyte-derived DCs (MoDCs) differentiated in vitro, remain mostly unsuccessful, only resulting in 5–15% objective clinical responses in numerous clinical trials. To date, Provenge (Sipuleucel-T) remains the first and only “DC” cancer vaccine to be approved by FDA for treatment of castration-resistant prostate cancer in 2010 [35]. Despite mostly unsuccessful clinical trials, recent findings that cDC1s play a critical role in cross-priming tumor-specific CD8 T cells and in determining the anti-tumor efficacy of cancer immunotherapies [36,37,38,39,40], has reignited the efforts to further develop/refine DC-based vaccines either as monotherapies or combined therapies. One of the major obstacles for DC-based cancer vaccines is tumor-mediated immunosuppression, often targeting DC function in cross-priming leading to CD8 T cell tolerance (cross-tolerance) instead of immunity [5,10,11,41,42,43]. Vaccines with DC-derived exosomes (DCexos), which are superior in their resistance to tumor-mediated suppression, bioavailability, and biostability compared to DCs, have demonstrated better anti-tumor efficacy than DC-based vaccines in preclinical trials, and thus have emerged as the promising alternative cell-free therapeutic vaccines that could overcome the obstacles of DC vaccines [44]. This review will examine the results and limitations of clinical trials, recent development on DCexo research and the future of DCexos as viable cancer vaccines. For more detailed review on DC-based cancer vaccines, readers are hereby referred to many recent excellent reviews [6,7,8,33,34,45].

## 2. DC-Derived Exosomes and Their Function

**Figure 1:**
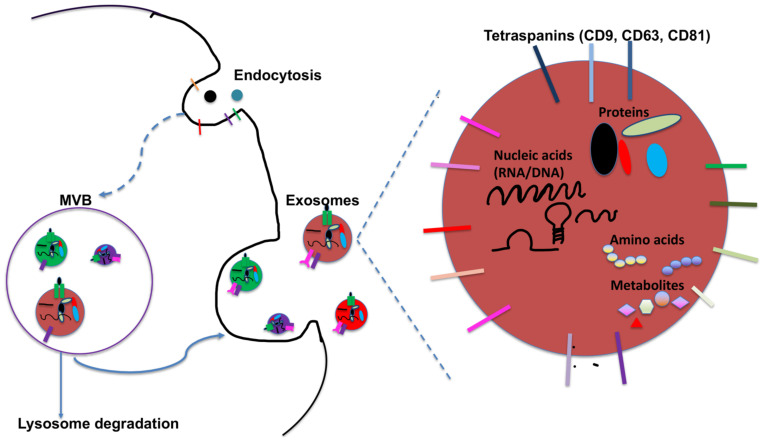
Biogenesis and characteristics of exosomes. Exosomes are produced in the endosomal compartment by inward budding of limiting endosomal membrane into intraluminal vesicles (ILVs) within the lumen of multivesicular bodies (MVBs). MVBs are either targeted for lysosomal degradation or they may fuse with plasma membrane to release these ILVs into the extracellular space as free exosomes. Exosomes are highly heterogenous with size ranging from 40 to 160 nm. Besides expressing an array of receptors on their surface, exosomes carry proteins, metabolites and nucleic acids including mRNA, miRNA, other non-coding RNA and DNA. Tetraspanins (CD9, CD63 and CD81) and other proteins such as Alix and TSG101 are often enriched in exosomes, and are commonly used as markers for exosomes.

All cells release extracellular vesicles (EVs) of different sizes and intracellular origin. These EVs could be broadly classified into three main groups according to their origin and size: the nanosized exosomes, microvesicles (MVs, also referred to as ectosomes), and apoptotic bodies that are constructed by direct sprouting of the cellular membrane in living and dying cells, respectively [46,47,48,49]. Apoptotic bodies are large vesicles generated by cells undergoing apoptosis, with 1000–5000 nm in size. MVs are generated through the direct budding or shedding from the plasma membrane by living cells, with a diameter from 50 to 1000 nm. On the other hand, exosomes are a heterogenous group of nano-sized EVs originating from endosomal pathway, with size ranging from 40 to 160 nm (Figure 1). Exosomes are produced in the endosomal compartment by inward budding of limiting endosomal membrane into intraluminal vesicles (ILVs) within the lumen of multivesicular bodies (MVBs, or so-called late endosomes) [48,49,50]. MVBs are either targeted for lysosomal degradation or they may fuse with the cellular membrane to release these ILVs into the extracellular space as free exosomes (Figure 1). Exosomes can contain membrane proteins, cytosolic and nuclear proteins, extracellular matrix proteins, metabolites, and nucleic acids including mRNA, miRNA, non-coding RNA, and DNA [49] (Figure 1). Although Exosomes were initially presumed to be an alternate route to excrete waste products in order to sustain cellular homeostasis, a seminal study have shown that exosomes carry and transfer mRNA and miRNA between cells [51]. It is well established now that exosomes play significant roles in intercellular communications and material transfer of their cargo [44,52,53]. In addition, exosomes are also no-immunogenic due to their similar membrane composition to the cells, biostable in vitro and in vivo, capable of targeting tissues and penetrating of biological barrier, making them attractive delivery vehicles for genetic material (miRNA, mRNA) and loaded drugs [54,55]. It should be noted that exosomes have great heterogeneity reflective of their size, content, function, and cellular origin, and current phenotypic and functional analyses of exosomes have been performed on only exosome-enriched populations, thus demanding caution when interpreting the data [49,55,56]. For more in-depth reading, several recent publications provided excellent comprehensive review on exosomes [46,47,48,49].

**Figure 2:**
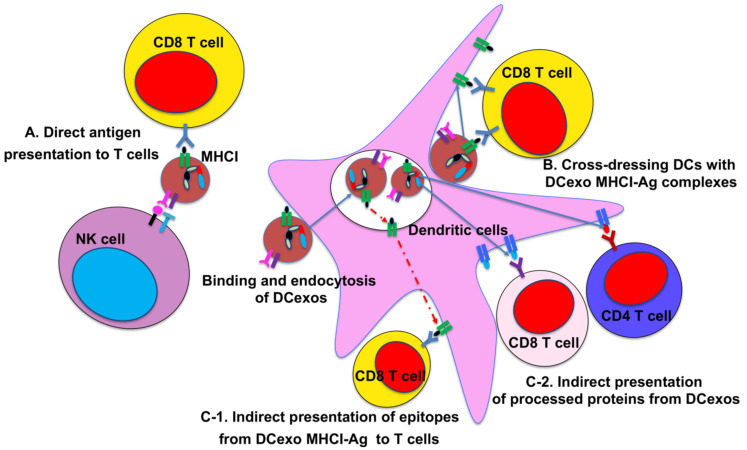
DCexo-mediated antigen presentation to activate T cells. (**A**): The presence of MHC_Exo_-Ag complexes on the surface of DCexos enables them to activate antigen-specific CD4 and CD8 T cells directly. Only MHCI and CD8 T cells are illustrated. NK cell-expressed ligands (NKG2D-L, IL-15R and BAG6) on DCexos can also activate natural killer (NK) cells directly. (**B**–**C**): DCexos activate antigen-specific T cells more efficiently indirectly via bystander DCs. (**B**): DCexos can transfer MHC_Exo_-Ag complexes to the DC plasma membrane, a process termed cross-dressing, leading to activation of antigen-specific T cells. MHC of DCexos and T cells has to be the same while MHC of the bystander DCs is not required. DCexos can also transfer MHC_Exo_-Ag complexes to tumor cells to be presented to host T cells (not depicted). (**C**): After internalization, (**C-1**) DCexos could transfer antigenic peptides to the MHC_DC_ in bystander DCs. The pMHC_DC_ complexes could be transported to the DC plasma membrane to be presented to T cells. DCexos, bystander DCs and T cells are required to have the same MHC in this mode. (**C-2**) Protein antigens carried by DCexos could be processed by bystander DCs, and multiple and different epitopes for both CD4 and CD8 T cells could be presented on MHC of bystander DCs. Only bystander DCs and T cells need to have the same MHC, allowing DCexos to induce allogeneic T cell responses.

DC-derived exosomes (DCexo) additionally carry functional MHCI and MHCII, co-stimulatory molecules (CD80, CD86) and adhesion molecules (ICAM1) involved in antigen uptake and presentation on their surface, which might be the most prominent feature distinct from exosomes produced from other immune cells [44,49,55,57]. As DCs process exogenous antigens in endosomal compartments including MVBs, which in turn fuse with plasma membrane resulting in the release of DCexos, DCexos have been shown to express both functional peptide/MHCI and peptide/MHCII complexes (pMHCI and pMHCII) for priming antigen-specific CD4 and CD8 T cells. It was first reported that exosomes generated from another APC--B cells express functional pMHCII complexes on their surface to activate antigen-specific CD4 T cells [58]. DCexos also carry high level of MHCI molecules, thus affording them the capacity to induce antigen-specific CD8 T cell responses [59]. Indeed, Zitvogel et al. have showed that DCexos from tumor-associated antigens (TAAs)–stimulated DCs prime tumor-specific CD8 T cell responses in vivo, and a single intradermal injection of DCexos achieved better anti-tumor efficacy in eradicating established tumors compared to the injection of DCs [60]. Although DCexos have been shown to directly prime T cells in vitro [59,61], this direct mechanism has been reported to be unable to prime naive T cells and is less likely to play a significant role in vivo [44,62] (Figure 2). Indeed, studies have shown that DCexos prime antigen-specific T cells far more efficiently with bystander DCs through indirect mechanisms. Several exosome membrane proteins, including integrins and ICAMs, facilitate their binding and subsequent internalization by bystander DCs, leading to indirect antigen presentation. One of the indirect antigens is called “cross-dressing”, referring to the direct transfer of exosomal pMHC complexes to the bystander DCs following exosome binding to bystander DCs (or other APCs) [44] (Figure 2). The second mechanism involves the processing and presentation of exosomal antigens onto MHC of bystander DCs, following binding and internalization of DCexos (Figure 2). In one scenario, DCexos pMHC_Exo_ complexes could be reprocessed through endosomal pathway in bystander DCs, resulting in the transfer of exosomal antigenic epitopes to bystander DC MHC molecules to be presented as pMHC_DC_ complexes on bystander DCs [44,63] (Figure 2). Alternatively, protein antigens carried by DCexos, which have been shown previously [64], could be processed by bystander DCs, and multiple different epitopes for both CD4 and CD8 T cells (or even B cells) could be presented on MHC_DC_ of bystander DCs (Figure 2). This mechanism might be most relevant for the use of DCexos as cancer vaccines, as studies have shown that only OVA protein-loaded but not peptide-loaded DCexos induced strong (allogeneic) CD8 T cell responses without requiring exosomal MHCI in vivo [65,66].

While DCexo-mediated T cell activation plays a critical function in their potential application in immunotherapy, DCexos also express NK receptors to induce NK cell activation [67] (Figure 2). In addition, DCexos have been shown to exert regulatory functions through exosomal membrane proteins or miRNA [68,69,70].

The capacity of DCexos to prime T cells—especially antigen-specific CD8 T cells—and their ability to shuttle different biomolecules, including proteins (such as antigens and cytokines) and RNAs to modulate immune responses, coupled with their amenability to modification of their composition and cargos, have presented DCexo-based vaccines as much improved alternative to DC cell-based vaccines [44,71,72,73,74]. Additionally, DCexos possess other advantages over DC(cell)-based vaccines. (1) DCexos have a more restricted and controllable molecular composition than DCs, owing to specific sorting and loading mechanisms. (2) DCexos have much longer shelf life than the very short shelf life of DCs. (3) DCexos can reach the proper location on secondary lymphoid organs more efficiently compared to DCs, and could be easily modified to deliver their cargos to specific targeted destinations [44]. (4) DCs are susceptible to tumor-mediated immunosuppression often observed in cancer patients, whereas DCexos being inert vesicles are not or more resistant. (5) DCexos might be more potent than DCs in activating T and NK cells, as DCexos actually present 10–100 times more pMHC complexes on their surface than DCs and have been shown to be enriched of activation ligands for NK cells [67,75]. Indeed, it has been reported that DCexos loaded with tumor antigens achieved better anti-tumor efficacy in eradicating established murine tumors compared to vaccines using DCs in preclinical models [60], thus supporting their clinical application as cancer vaccines [44,57,76,77,78].

## 3. DC-Derived Exosomes in Clinical Trials

Three clinical trials using DCexos including two phase I and one phase II clinical trials (see Table 1) have been completed [79,80,81]. In addition, there was one phase I clinical trial, which treated advanced colorectal carcinoma (CRC) patients with autologous ascites-derived exosomes (ASexos) either alone or in combination with GM-CSF [82]. ASexos were prepared from ascites of the CRC patients, that were enriched for MHCI and MHCII, HSPs (including HSC70, HSP70 and HSP90), CD80 and ICAM1. While ASexos were mainly derived from CRC cells in the ascites, they likely also contained exosomes from immune cells including DCs.

## 4. DCexo Phase I Clinical Trials

The first phase I clinical trial with DCexos as cancer vaccines employed DCexos obtained from autologous immature monocyte-derived DCs (MoDCs) (Table 1). Exosomes were isolated by ultracentrifugation and loaded with both MHCI and MHCII melanoma-associated antigen (MAGE) peptide epitopes. An MHCI-restricted cytomegalovirus (CMV) peptide and an MHCII-restricted tetanus toxoid-derived peptide were also loaded onto exosomes. Peptides were loaded either directly onto isolated exosomes, or indirectly by adding peptides into DC culture that produced exosomes, and the antigen-loaded exosomes were then administered into advanced non-small cell lung cancer (NSCLC) patients [79]. A total of 13 HLA-A2^+^ patients with pre-treated advanced stage (IIIb and IV) NSCLC were enrolled, and 9 patients completed therapy after receiving 4 exosome doses at weekly interval. Only grade 1–2 toxicity and no autoimmune reactions were observed, suggesting that the exosome vaccine was safe and well-tolerated in patients similar to DC vaccines. One week after the last DCexo injection, three patients of the tested participants, who had not shown MAGE-specific immune responses before DCexo injections, exhibited systemic MAGE-specific immune reactivity as measured by delayed- type hypersensitivity (DTH) response. Increased MAGE-specific T cell responses were only observed in one of five patients examined by enzyme-linked immunospot (ELISPOT) assay. However, no antigen (MAGE)-specific T cell responses were observed in PBMCs by in-vitro assays. The low rate of T cell activation was attributed to potential suppression by regulatory T cells (Tregs, CD4^+^CD25^+^ T cells). In two of three patients examined, the percentages of Tregs out of total CD4^+^ T cells were increased following DCexo vaccinations. Interestingly, two of four tested samples exhibited increased NK cell lysis activity. Overall, the NSCLC phase I study showed that DCexo vaccines were well-tolerated with an acceptable safety profile, with disease stability observed in two patients who had progressive cancer at diagnosis. In addition, disease stability continued for over 12 months in two of four patients with stable disease [79].

The other DCexo phase I clinical trial enrolled 15 MAGE3^+^ advanced (stage IIIb or IV) metastatic melanoma (MM) patients (Table 1). Exosomes were isolated by ultracentrifugation from autologous immature MoDCs, and loaded with both MHCI and MHCII MAGE3 peptide epitopes either directly or indirectly. Similar to the NSCLC trial, an MHCII-restricted tetanus toxoid-derived peptide was also loaded unto DCexos. All patients were administered 4 doses of DCexos at a weekly interval, and evaluation of the vaccine efficacy was conducted two weeks after vaccinations. Of these patients, one patient exhibited a partial response to DCexo immunotherapy. In this patient, a halo of depigmentation around naevi was observed, and the arterial neovasculature disappeared and tumor size reduced. This patient received 4 months of continuation therapy with DCexos, leading to disease stabilization and reduced toxicity. Stabilization of the disease for up to 24 months was also observed in another patient who was given continued DCexo immunotherapy. Overall, this clinical trial resulted in two stable diseases, as well as one minor, one partial, and one mixed response at lymph nodes or skin. Some of these responses were achieved in patients with progressive disease who had formerly been given other cancer therapies. Similar to the NSCLC phase I clinical trial, neither DTH responses or MAGE-specific T cell responses were observed in peripheral blood, although T cell responses against MART1 (melanoma antigen recognized by T cells 1) which were not included in the vaccines, were detected [80]. In contrast, NK cell effector functions were enhanced in peripheral blood of 8/13 patients following DCexo vaccination [80], thus suggesting that augmented NK cell functions in vivo might account for the T cell-independent clinical responses.

As enhanced NK cell activation was observed in the two phase I clinical trials, Viaud S. et al. further examined whether NK cell activation instead of T cells could be responsible for the clinical effects observed in the clinical trial carried out by Escudier B. et al. [67]. Indeed, exosomes generated from immature human DCs express killer cell lectin–like receptor subfamily K, member 1 ligands (NKG2D-L), which can directly interact with NKG2D on NK cells, resulting in their activation [67]. Examining samples from the DCexo clinical trial on MM [80], Viaud S. et al. observed that circulating NK cell numbers were significantly increased after 4 DCexo vaccinations. Moreover, the expression of NKG2D and NK cytotoxicity were restored after vaccinations in 50% of patients who had NK cell function defects at the beginning of the clinical trial [67]. Further studies have shown that DCexo vaccinations induce NK cell proliferation in an IL-15Rα–dependent manner. These findings on DCexo-mediated effects on NK cells are consistent with improved control of tumor metastasis in B16F10-bearing C57BL/6 mice by NK1.1^+^ cells [67]. Interestingly, exosomes generated from human immature DCs have also been reported to express BCL2-associated athanogene 6 (BAG6, also known as BAT3), a ligand for NKp30 receptors expressed on NK cells [83,84]. Cytokine production of NK cells has been reported to directly correlate with exosomal BAG6 expression levels l [84]. Additionally, DCexo expression of TNF superfamily ligands TNF, FasL, and TRAIL on their surface activate NK cells and stimulate them to secrete IFN-γ [69]. Taken together, the two phase I clinical trials and follow-up studies suggest that DCexos likely possess the capability to activate NK cells to generate anti-tumor immunity.

The third phase I clinical trial that might involve DCexos used ascites-derived exosomes (ASexos) alone or in combination with GM-CSF to treat 40 advanced colorectal carcinoma (CRC) patients [82] (Table 1). Exosomes were prepared from patient ascites, and were shown to be enriched in MHCI and MHCII, CD80, and ICAM1 similar to DCexos. In addition, these ASexos also contained the immunogenic carcinoembryonic antigen (CEA) of CRC. The patients received 4 weekly immunizations. Unlike the other two phase I clinical studies, DTH responses as well as CEA-specific CTL cell responses were observed in patients treated with ASexos plus GM-CSF. A higher level of tumor-associated antigens (TAAs) in ASexos may be responsible for the augmented T cell responses compared to the two aforementioned DCexo phase I trials. Despite the T cell responses, however, no detectable therapeutic responses were observed with the exception of one stable disease and a minor response after ASexos plus GM-CSF treatment. Another drawback for this model is that the majority of the ASexos were likely derived from the CRC cells instead of immune cells including DCs, and tumor-derived exosomes have been shown to be often immunosuppressive and promote tumor growth, metastasis, and development of drug resistance [85,86].

## 5. DCexo Phase II Clinical Trial

The limited clinical benefit shown by the phase I trials using exosomes from immature MoDCs prompted researchers to design and develop innovative strategies to promote DCexo-induced anti-tumor host immune responses. Based on previous studies showing that DCexos from mature DCs prime T cells more efficiently compared to DCexos from immature DCs [61,87], one strategy is to utilize DCexos originated from matured DCs. A clinical-grade process for producing DCexo vaccines was developed with human DC cultures [88]. Here, IFNγ was employed to stimulate human MoDCs in culture, and subsequently, costimulatory factors and ICAMs were upregulated, resulting in second-generation DCexos (IFNγ DCexos) with increased immunostimulatory capacity [88]. A phase II clinical trial was carried out with these second-generation IFNγ DCexos, aiming to investigate whether maintenance immunotherapy of advanced NSCLC patients using IFNγ–DCexos could increase progression-free survival (PFS) at 4 months following platinum-based chemotherapy [81] (Table 1). Twenty-two advanced HLA-A2^+^ NSCLC patients who had inoperable (stage IIIb or IV) NSCLC with neutrophils ≥1.5 × 10^9^/L and showed immune responses or disease stabilization following 4 rounds of a first-line platinum-based chemotherapy were eligible to receive IFNγ DCexos [81]. Both MHCI-restricted (MAGE-A1, MAGE-A3, NY-ESO, MART1) and MHCII-restricted (EBV) TAA were used. Patients first received 3 weeks of metronomic oral low-dose cyclophosphamide (CTX) prior to IFNγ DCexo maintenance therapy, based on both preclinical and clinical data showing that this protocol reduces Treg function and induces IFN-γ/IL-17–producing T cells [89,90,91,92]. Of these participants, 7 patients (32%) exhibited stable disease after 9 times of IFNγ DCexo vaccinations, and proceeded to receive DCexo therapy every 3 weeks. Unfortunately, a PFS of 50%, the primary end-point of the trial, was not reached, and no objective response was reported in the clinical trial. However, one patient exhibited a long-term disease stabilization, which allowed for surgical removal of the tumor and the eligibility for local adjuvant radiotherapy.

As to immunological readouts, the use of IFNγ DCexos as cancer immunotherapy were again insufficient to induce TAA-specific T cell responses in patients despite loading of multiple epitopes and CTX adjuvant therapy [81]. Thus, the immunostimulatory effects by IFNγ DCexos was likely mediated via augmented NK cell activation through NKp30 signaling. Indeed, increase in NKp30-mediated IFNγ and TNFα production by circulating NK cells was observed upon four IFNγ DCexo vaccinations, although NK cells in these advanced NSCLC patients only exhibited low levels of NKp30. More significantly, this increased NKp30-elicited NK cell activation correlated with longer PFS. In addition, the membrane-associated NKp30 ligand, BAG6, was detected on the membrane of DCexo vaccine preparations and was reported to play a critical role in mediating NK cell activation through a NKp30-dependent manner, thus supporting IFNγ DCexos promote NK cell activation/function through a NKp30-dependent mechanism. Moreover, BAG6 levels correlated with the MHCII concentrations of DCexos and NKp30-dependent NK cell activation. It should be noted that the NKp30-dependent NK activation differs from the finding of the phase I clinical trial on MM where NKG2D/NKG2D-L (and potentially IL-15/IL-15Rα) signaling mediated DCexo-induced NK activation [67,80]. Given that the DCexos employed in the MM clinical trial were not generated from MoDCs matured by IFNγ, which has been shown to upregulate BAG6, NKG2D/NKG2D-L–mediated NK cell activation likely plays a more prominent role instead of NKp30/BAG6 signaling.

Overall, these DCexo phase I and II clinical trials have demonstrated that DCexo vaccines are well-tolerated and safe, and are amenable to large-scale production in clinical settings. While only partial or minor responses were observed in these clinical trials with small number of patients, some patients achieved stabilization of disease.

While we focused our discussion on DCexos, it’s worth pointing out that exosomes from tumor cells, mesenchymal stem cells, and other immune cells such as B cells, macrophages, and NK cells, T cells have also been examined for their application in cancer immunotherapy [55,62]. Indeed, tumor cells were likely the main source of the ASexos in one of the clinical trials we mentioned above [82]. Although tumor cell-derived exosomes (Texos) are capable of inducing anti-tumor immune responses, Texos generally seem to exhibit immune-suppressive functions [55,62]. Nevertheless, vaccines with Texos have emerged as promising cancer vaccines, likely due to their enrichment of tumor antigens making them capable of inducing T and B cell responses [93]. One promising approach to counter the suppressive properties of these exosomes is exosome engineering—to modify surface molecules on exosomes to induce tumor cell death or improve targeted delivery of exosomal cargos, to modify exosomal contents to deliver miRNA, mRNA, and cytokines for immune modulation, thus improving their efficacy [72,73]. The application of these exosomes was excellently reviewed recently [47,86,93,94].

## 6. Conclusions and Future Directions

The three clinical trials of DCexos as cancer vaccines have shown limited clinical efficacy in advanced cancer patients, which could be attributed to weak induction of adaptive immune responses specifically T cell responses in these patients. The poor adaptive immune responses could be potentially due to several factors: (1) the heterogeneity and the limited number of the patients, who had received previous treatments before enrollment; (2) systemic and local immunosuppressive mechanisms often present in these advanced-stage patients; (3) lack of sufficient maturation/adjuvant signals; (4) autologous MoDCs might not be the best DCs to achieve the optimal anti-tumor T cell responses; and (5) T cell antigens employed in these clinical trials might be insufficient to induce tumor antigen-specific T cell responses [44,63].

Given the low clinical efficacy and lack of antigen-specific T cell responses in all clinical trials with DCexos, there is a critical need to develop strategies to augment DCexo functions in generating anti-tumor T cell immunity to improve the anti-tumor efficacy of DCexo vaccines. A number of approaches have been discussed in detail in several reviews recently [44,63,95]. Briefly, the following improvements have been proposed: (1) Based on the phase II trial data on NSCLC [81], DCexo immunotherapy was likely most effective in patients with measurable levels of serum BAG6, which is possibly related to NKp30 functional defects. Thus, selection of patients who showed downregulation or defective functions of NK receptors (particularly NKG2D or NKp30), will likely improve the efficacy of DCexo therapy. The screening of NK receptors in the patients can be achieved now by using high-dimensional immunoprofiling approach such as CyTOF [96]. Along the same line, to generate synergistic immunogenic effects against NK-dependent cancers including gastrointestinal stromal tumors, neuroblastomas, and kidney cancers, DCexo vaccines could be combined with NK-based therapies, such as anti-KIR Ab (anti-killer cell immunoglobulin-like receptor antibody) [97,98,99]. (2) To counter systemic or local immunosuppressive mechanisms often observed in patients with advanced cancers, DCexo vaccines could be combined with other therapy regimes that reduce tumor-mediated immunosuppression. For example, in the phase II clinical trial DCexo vaccines were combined with CTX treatment [81], which has been shown in preclinical and clinical studies to reduce Treg function and stimulate dual IFN-γ/IL-17–producing T cells [89,90,91,92]. Unfortunately, objective responses were not observed in the Phase II NSCLC clinical trial even with the combination treatment, likely due to the advanced stages of NSCLC. However, combination treatments with other immunotherapies including immune checkpoint blockade (ICB) remain promising approaches. (3) To employ additional TLR ligand adjuvants as DC maturation signals, as DCexo-induced anti-tumor immune response directly depends on the degree of maturity of DCs and the type of maturation stimuli. For example, DCexos from DCs treated with poly(I:C) have been shown to be the most efficient in a model of B16-OVA melanoma in vivo compared to other TLR ligands, inducing robust activation of melanoma-specific CD8 T cells in tumor-draining lymph nodes, spleen, and tumor tissues and recruited NK and NKT cells to the tumor site, resulting in drastic inhibition of tumor growth and an increase in survival in tumor-bearing animals [100]. Together with other studies, TLR3 ligand poly(I:C) seems to be a favorable TLR agonist for DC maturation during antigen loading, which significantly increased the potential for DCexo-induced anti-tumor immunity, and could be employed as a promising maturation stimulus to generate DCexos in future clinical trials. (4) In addition, DCexos could be engineered to improve their migration and immunostimulatory capacity. DCexos could be modified to express TNF, FasL, and TRAIL to target tumor cells directly and induce tumor cell apoptosis, and DCexos could be engineered to transfer miRNAs, cytokines, and chemokines, mRNAs that encode relevant neoantigens or regulatory proteins to modulate gene functions in targeted immune cells or cancer cells. Similarly, immortalized DC cell lines, which could bypass the demanding procedure of generating autologous MoDCs on advanced cancer patients, could be amendable to generate DCexos of desired modification. (5) For DC vaccines, one promising approach to overcome the functional limitations of autologous MoDCs used in all three DCexo clinical trials is to use naturally circulating primary DCs (nDCs) [45,101]. Indeed, several clinical trials using naturally circulating DCs including cDC2s and/or pDCs have shown that nDC vaccines are safe and well-tolerated in patients, with the induction of antigen-specific immunity in some patients [102,103,104,105,106,107]. Conceivably, corresponding exosomes generated from theses DCs could be tested as vaccines. Similarly, exosomes from immortalized DC cell lines such as the human pDC cell line used in GeniusVac-Mel4 clinical trial) would bypass the need of using autologous MoDCs [108]. (6) To expand TAAs beyond the current T cell-restricted epitopes to augment anti-tumor adaptive immune responses, as recent studies have suggested that both B cells and CD4 T cells played critical role in DCexo-induced antigen-specific CD8 T cell responses [64,65]. In addition, the same group has shown that DCexos loaded with protein antigens but not with peptide antigens were capable of inducing allogeneic CD8 T cell responses, leading to the suggestion that allogeneic DCexos should be tested as cancer vaccines [66].

It should be noted that the two major presumed advantages for DCexo vaccines over DC-based vaccines; namely, better anti-tumor efficacy and resistance to immunosuppression, have not been realized in the three DCexo clinical trials [79,80,81]. While these strategies discussed above will undoubtedly improve on current DCexo-based cancer vaccines, they are unlikely to overcome the hurdles to move DCexo vaccines forward as discussed below. One of the major drawbacks of the DCexo clinical trials is that all three current DCexo clinical trials use peptide-pulsed DCexos from labor- and cost-intensive autologous MoDCs, based on the idea that exosomal pMHC complexes play a critical role in priming T cells. MoDCs were generated from autologous DC culture systems [44], where a leukapheresis is performed on already immunocompromised advanced cancer patients. The patient loses important immune cells, and the cells may be suboptimal. Indeed, ex vivo differentiated MoDCs have been shown to differ from the primary DCs both in phenotypic and transcriptional features and are less efficient in migratory capacity and T cell activation [95]. All three DCexo clinical trials, however, have shown limited clinical efficacy and induced little or no antigen-specific T cell responses, although enhanced NK cell activity was observed, which likely contributed to the improved clinical outcomes [79,80,81]. Collectively, these data suggest that exosomal pMHC complexes on peptide-pulsed DCexos from autologous MoDCs are likely not sufficient and/or critical to prime T cells in vivo, consistent with recent report showing that protein-loaded DCexos but not peptide-loaded DCexos induced antigen-specific T cell responses in vivo [65]. However, protein-loaded DCexos have not been tested in clinical settings. Given that current DCexo studies are focusing only on peptide- or protein-loaded DCexos from ex vivo differentiated MoDCs [44,109], there is an urgent need to expand our studies on DCexos beyond MoDCs, to develop new approaches to generate DCexos that are able to prime (CD8) T cells and generate anti-tumor immunity in vivo.

Several developments support/demand the expansion of DCexos from other DCs such as other DC subsets and primary DCs. For example, clinical trials with naturally circulating primary DCs including CD1c^+^ conventional type 2 DCs (cDC2s) and plasmacytoid DCs (pDCs) are well-tolerated and safe in patients with promising results [103,104,107]. A new report on previously unreported pDCexos offers an important and exciting addition to current arsenal of DCexos [110], which we will discuss in more details.

## 7. Plasmacytoid DC-Derived Exosomes—The New Addition to DCexos

Although pDCs were generally thought to play a tolerogenic role in tumors as accumulation of pDCs in multiple tumors was often associated with poor prognosis [20,21,111,112,113], immunotherapies with pDCs either alone or in combination with cDCs have shown promising clinical results [45,101,103,114,115]. However, it remains unclear whether pDCs exert their effects directly through their cross-priming or indirectly by regulating other immune cells (i.e., cDCs, regulatory T cells, and NK cells) through pDC activation and subsequent production of IFNI and other cytokines [116,117]. In fact, even the involvement of pDCs in cross-priming in vivo is still under debate [118,119,120,121], although pDCs have been shown to be capable of cross-presentation in vitro [122,123,124,125,126]. Moreover, whether pDCs generate exosomes have not been investigated, although earlier studies have shown that exosomes could regulate the functions of pDCs [127,128,129].

As it remains unclear how pDCs exert their functions in inducing anti-tumor CD8 T cell immunity or promoting tolerance, our group decided to use a pDC-targeted vaccine model to investigate how pDCs achieve cross-priming of antigen-specific CD8 T cells [110]. Previous studies have shown that pDC-targeted anti-Siglec-H and anti-Bst2 antibodies delivered antigens to only pDCs, but not cDCs in vivo [130,131]. As anti-Siglec-H-Ag have been reported to induce CD4 T cell tolerance with or without adjuvants [130], we first employed pDC-targeted anti-Siglec-H-OVA to investigate if pDCs similarly cross-prime CD8 T cells to induce tolerance in vivo. To our surprise, vaccination with anti-Siglec-H-OVA plus CpG as adjuvant resulted in strong cross-priming of OVA-specific CD8 T (OTI) cells and recalled memory CD8 T cell responses [110]. Interestingly, pDC-mediated cross-priming in vivo is dependent on non-targeted cDCs, as depletion of cDCs abrogated effector differentiation of antigen-specific CD8 T cells [110]. Further analysis revealed that while pDCs transferred antigens to cDCs leading to both pDCs and cDCs expressing MHCI-antigen (pMHCI, H-2K^b^-SIINFEKL) complexes on their surfaces, only cDCs but not pDCs effectively primed naive OTI cells ex vivo, suggesting that pDCs likely achieve cross-priming by transferring antigens to non-targeted cDCs [110]. Taking advantage of an in vitro culture system where antigens were only accessible to pDCs, we were able to confirm the requirement of bystander cDCs for pDC-mediated cross-priming, showing that cross-presenting pDCs primed naive CD8 T cells by transferring antigens to bystander cDCs [110]. Thus, our data suggest that cross-presenting pDCs transferred antigens to naïve bystander cDCs, thus conferring bystander cDCs the ability to cross-priming CD8 T cells [110].

We next investigated how cross-presenting pDCs transferred the antigens to cDCs. Using multiple approaches, we have further demonstrated that cross-presenting pDCs transferred antigens to bystander cDCs through pDC-derived exosomes (pDCexos). Interestingly, pDCexo-mediated priming of CD8 T cells was dependent on the presence of bystander cDCs, similar to cross-presenting pDCs, suggesting that cross-presenting pDCs achieve cross-priming through a novel mechanism of pDCexo-mediated antigen transfer to cDCs [110]. The pDCexo-mediated antigen transfer to cDCs is not limited to targeting pDCs via Siglec-H. Using both soluble proteins and antigens targeted to pDCs through anti-Bst2, we further showed that pDCs loaded with soluble proteins or antigens delivered through anti-Bst2 generated pDCexos, which similarly induced cDC-dependent cross-priming by transferring antigens to bystander cDCs. Taken together, our data has suggested that pDCs employ an exosome-mediated and cDC-dependent mechanism for cross-priming under multiple settings [110].

## 8. The Future of DCexos as Cancer Vaccines?

The identification of previously unreported pDCexos not only provides an interesting addition to current DCexo arsenal, but also open up new avenues for expanding DCexo research. As pDCexos function similarly to their counterpart pDCs, it’s worthy to explore exosomes from different in-vitro differentiated DCs, as well as primary DCs to determine their potential in cancer vaccines [102,103,104,105,106,107,132].

As multiple clinical trials of pDC-based vaccines have already reported promising results [103,108,115], it is conceivable that cancer vaccines with pDCexos could combine the advantages of both pDC and DCexo vaccines. Compared to pDCs, pDCexos are more resistant to tumor-mediated immunosuppression and more biostable, and thus might achieve better anti-tumor efficacy. More excitingly, a pDC vaccine clinical trial using a human pDC cell line (GeniusVac-Mel4 clinical trial) has shown promising results [108]. The availability of multiple well-characterized human pDC cell lines, including the one used in GeniusVac-Mel4 clinical trial [108,133,134,135], will in theory produce pDCexos without quantity limitation at low cost, and eliminate the need of the demanding leukapheresis on vaccine patients often with advanced cancers. Production of pDCexos from these immortalized pDC cell lines will also reduce production time and is more amendable to quality control and scale up. Further studies on these pDCexos are warranted to determine their potential clinical application in cancer immunotherapy.

On the other hand, the use of DC-targeted antibody carrying desired antigens to generate pDCexos also opens up the field beyond the current peptide- or protein-loaded DCexos [44,109]. As cDC1-targeted anti-DEC-205-antigen has been shown to be about 1000 times more efficient in cross-presentation compared to soluble protein antigens [136], it would be interesting to investigate if pDCexos loaded with pDC-targeted antigens are also more efficient in cross-priming than pDCexos loaded with non-targeted protein antigens. Along the same line, our identification of pDCexos loaded with pDC-targeted antigens prompted us to ask if we could similarly generate cDC-derived exosomes (cDCexos) using cDC-targeted antigens such as anti-DEC-205-Ag, and whether such exosomes function more efficiently in cross-priming than cDCexos loaded with peptide antigens or non-targeted protein antigens. Of note, human anti-DEC-205 carrying NY-ESO-1 targeting DEC205-expressing cDCs induced both humoral and NY-ESO-1-specific CD4 and CD8 T cell responses, and achieved partial clinical responses in a phase I clinical trial [137]. Studies are warranted to further investigate how these pDCexos and/or cDCexos loaded with DC-targeted antigens function in vivo to determine if these DCexos are suitable as cancer vaccines.

Another related question raised from our pDCexo study is how pDCexos generated with pDC-targeted antigens transfer antigens to cDCs to achieve CD8 T cell priming. Interestingly, Gabrielsson’s group has reported recently that DCexos generated with soluble OVA protein carried intact OVA protein [64], and these OVA-loaded DCexos induced strong allogeneic CD8 T cell responses requiring no exosomal MHC [65,66]. As uptake of pDC-targeted antigens was mediated by receptor-mediated endocytosis similar to soluble OVA protein [138], pDCexos might similarly carry intact antigens to be transferred to bystander cDCs. Indeed, our preliminary data have shown that pDCexos loaded with pDC-targeted anti-Siglec-H-Ag carried the intact anti-Siglec-H-Ag and efficiently primed allogeneic CD8 T cells in vitro and in vivo [139]. Future studies are warrantied to determine if pDCexos could be employed as impersonalized vaccines that are broadly applicable without MHC restriction [63]. Together with the potential of generating pDCexos from available human pDC cell lines, one of which has already been employed in a clinical trial with promising results [108], we would argue that pDCexos might represent the most promising DCexo candidate as cancer vaccines that could overcome the hurdles presented in previous DCexo clinical trials.

**Figure 3:**
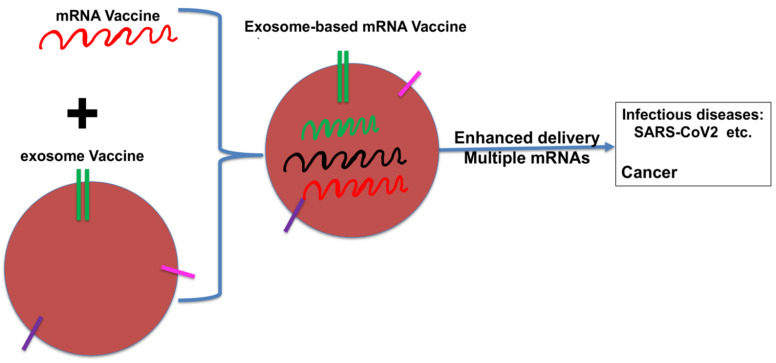
Exosome-based mRNA vaccines for immunotherapy. Combining exosome and mRNA vaccines might represent a promising strategy to further improve mRNA-based vaccines again infectious diseases and cancer: receptors on exosomes could enhance the targeted delivery of mRNAs, and multiple mRNAs could be efficiently delivered.

While we are focused on the application of exosomes in cancer immunotherapy, exosome-based vaccines have also emerged as good candidates for rapid development of vaccines against infectious diseases due to their increased efficacy and versatility [71]. Cross-talk between their applications in cancers and infectious diseases will likely benefit both fields. Indeed, a casual search will find that at least 7 registered human clinical trials are testing exosomes/EVs as therapeutics for treating severe acute respiratory syndrome coronavirus 2 (SARS-CoV2) (ClinicalTrials.gov Identifier: NCT04276987, ClinicalTrials.gov Identifier: NCT4384445, ClinicalTrials.gov Identifier: NCT04389385, ClinicalTrials.gov Identifier: NCT04491240, ClinicalTrials.gov Identifier: NCT04493242, ClinicalTrials.gov Identifier: NCT04602442 and ClinicalTrials.gov Identifier: NCT04798716). Our recent study has shown that resting primary HPBCs harbor abundant cytoplasmic angiotensin-converting enzyme 2 (ACE2) protein and that circulating exosomes contain ACE2, the surface expression of which is indispensable for SARS-CoV2 infection of circulating monocytes/macrophages [140], highlighting the potential of exosome-based and/or exosome-targeted immunotherapies against COVID-19. Furthermore, exosome-based vaccines might potentially synergize with mRNA-based vaccines, which have shown much success in generating efficient vaccines against SARS-CoV2 [141]. Indeed, mRNA-based vaccines are revolutionizing the field of rapid development of vaccines for emerging pathogens and have reported encouraging data in personalized neoantigen vaccines using mRNAs encoding neoantigens of patients [142,143], although achieving an efficient cytoplasmic delivery of mRNA to target cells remains one major challenge. Exosomes/EVs are known to play a critical role in intercellular communication, shuttling proteins, metabolites and nucleic acids including mRNA, miRNA, non-coding RNA, and DNA [47,49,78]. While exosomes have not been tested as potential vehicles in mRNA vaccination, exosomes have been reported to be excellent vehicles to transport mRNAs and to target the delivery to secondary lymphoid organs efficiently [144]. More importantly, direct application of mRNA or its electroporation into DCs was shown to induce polyclonal antigen-specific CD4 and CD8 T cell responses as well as the production of protective antibodies with the ability to eliminate transformed or infected cells [141]. More than 10 clinical trials on mRNA vaccines using DCs as vehicles have been completed, with similar number of ongoing clinical trials [141]. Given that exosomes are amendable to modification by introducing exogenous cargos into or unto exosomes, either through direct modification or manipulation of the parental cells [71,72,73,74], exosomes could serve as suitable vehicles for delivering mRNAs as vaccines. Similarly, DCexos would be easily modified to carry multiple desired mRNAs to augment anti-tumor immune responses and improve the anti-tumor efficacy of mRNA-based cancer vaccines. Thus, combining exosome-based and mRNA-based vaccines might represent a promising strategy to further improve mRNA-based vaccines again infectious diseases and cancer (Figure 3).

Despite the great promise of DCexo-based immunotherapy, the advance of DCexos as cancer vaccines has stalled due to the limited clinical efficacy of recent clinical trials [79,80,81]. However, the expansion of DCexos beyond the peptide-loaded DCexos from MoDCs, coupled with recent advance in exosome-based therapies against COVID-19 and their potential use in combination with mRNA-based vaccines, suggest that the future for exosomes/DCexos as cancer vaccines is very bright.

## Figures and Tables

**Table 1:** Summary of current clinical trials with DC-derived exosomes (DCexos).

Cancer Type	Phase	Exosomes /Antigen	Doses	Patients	Toxicity	Clinical Outcomes
Advanced Non-small cell lung cancer	I	Exosomes were isolated from autologous MoDCs generated in vitro, and loaded with MAGE peptides	once weekly for 4 weeks	13 (9 completed) HLA-A2^+^ stage IIIb and IV NSCLC patients with tumor expression of MAGE3 or MAGE4	Grade 1–2 toxicity	DTH reactivity against MAGE peptides in 3/9; MAGE-specific T cell responses in 1/3 patients examined; increased NK lytic activity in 2/4 [79].
MAGE3- expressing advanced melanoma	I	Autologous MoDC-derived exosomes were loaded with MAGE3 peptides	once weekly for 4 weeks	15 stage IIIb and IV, HLA-A1^+^, B35^+^ or HLA-DPO4^+^ patients	Only grade 1 toxicity	No detectable MAGE3-specific CD4 and CD8 T cell responses; restored NKG2D expression and NKG2D-dependent function of NK cells in 7/14 patients; 1/15 partial responses [67,80].
Advanced colorectal cancer	I	Exosomes from patient ascites + GM-CSF, ASexos contained CEA with no additional antigen loading.	once weekly for 4 weeks	40 HLA-A2^+^CEA^+^ stage III and IV CRC patients	Grade 1–2 toxicity	DTH induction in both groups, and CEA-specific CTL responses were observed in ASexo + GM-CSF group. 1 stable disease and 1 minor response in ASexo + GM-CSF group [82].
Non-small cell lung cancer	II	IFN-γ-matured autologous MoDCs were loaded with both MHCI and MHCII tumor epitopes.	exosome immunization in 1, 2 and 3 week intervals in a maintenance immunotherapy regime	26 (22 HLA-A2^+^ stage IIIb and IV NSCLC patients	1/22 grade 3 hepato-toxicity	No detectable induction of antigen-specific T cell responses; increased NKp30-dependent NK cell function; 7 patients (32%) with progression-free survival at 4 months after chemotherapy cessation; no objective tumor response according to RECIST criteria [81].

## Data Availability

The data presented in this study is available within the article.
